# A Systematic Review of Characteristics Associated with COVID-19 in Children with Typical Presentation and with Multisystem Inflammatory Syndrome

**DOI:** 10.3390/ijerph18168269

**Published:** 2021-08-04

**Authors:** Jeffrey Kornitzer, Jacklyn Johnson, Max Yang, Keith W. Pecor, Nicholas Cohen, Carolyn Jiang, Xue Ming

**Affiliations:** 1 Division of Neurology, New Jersey Pediatric Neuroscience Institute (NJPNI), Morristown, NJ 07960, USA; jkornitzer@njpni.com; 2 Department of Neurology, Rutgers New Jersey Medical School, Newark, NJ 07103, USA; jaj176@njms.rutgers.edu; 3 College of Arts and Sciences, University of Pennsylvania, Philadelphia, PA 19104, USA; maxyang@sas.upenn.edu (M.Y.); nicholas.cohen9@gmail.com (N.C.); 4 Department of Biology, The College of New Jersey, Ewing, NJ 08628, USA; pecor@tcnj.edu; 5 School of Arts and Sciences, Rutgers University, New Brunswick, NJ 08901, USA; cantertohappyness@gmail.com

**Keywords:** Kawasaki-like, hyperinflammation, anosmia, lymphocytopenia, SARS-CoV-2, MIS-C

## Abstract

Setting off a global pandemic, coronavirus disease 2019 (COVID-19) has been marked by a heterogeneous clinical presentation that runs the gamut from asymptomatic to severe and fatal. Although less lethal in children than adults, COVID-19 has nonetheless afflicted the pediatric population. This systematic review used clinical information from published literature to assess the spectrum of COVID-19 presentation in children, with special emphasis on characteristics associated with multisystem inflammatory syndrome (MIS-C). An electronic literature search for English and Chinese language articles in COVIDSeer, MEDLINE, and PubMed from 1 January 2020 through 1 March 2021 returned 579 records, of which 54 were included for full evaluation. Out of the total 4811 patients, 543 (11.29%) exhibited MIS-C. The most common symptoms across all children were fever and sore throat. Children presenting with MIS-C were less likely to exhibit sore throat and respiratory symptoms (i.e., cough, shortness of breath) compared to children without MIS-C. Inflammatory (e.g., rash, fever, and weakness) and gastrointestinal (e.g., nausea/vomiting and diarrhea) symptoms were present to a greater extent in children with both COVID-19 and MIS-C, suggesting that children testing positive for COVID-19 and exhibiting such symptoms should be evaluated for MIS-C.

## 1. Introduction

A newly identified strain of coronavirus, severe acute respiratory syndrome coronavirus 2 (SARS-CoV-2), is a highly contagious cause of a systemic disease known as coronavirus disease 2019 (COVID-19). In adults, COVID-19 commonly presents as viral pneumonia with gastrointestinal disturbances, ageusia, and anosmia [1]. As of 14 May 2021, the virus has infected over 160,800,000 individuals and led to over 3,300,000 deaths [2]. However, even with multiple waves of the pandemic and variants that affect different regions of the world, there is still a lack of understanding of COVID-19, especially in pediatric populations.

According to the United States Centers for Disease Control and Prevention, children and adolescents with COVID-19 are more likely to have mild, non-specific symptoms or be asymptomatic compared to adults [3]. However, asymptomatic individuals and those with mild symptoms are still able to transmit the virus, thus it is vital to understand the manifestations of COVID-19 in children, as they still pose a risk to more vulnerable populations. Furthermore, although children and adolescents are less likely to develop serious illness and die due to COVID-19, 340 related deaths among persons ages 0–18 have been reported to the National Center for Health Statistics through 12 May 2021 [4]. Although rates of severe outcomes from COVID-19 including mortality and hospitalization in school-aged children are low, severe complications are more commonly reported in children with underlying medical conditions.

Owing to a lack of sufficient experience and unified pediatric population-based data from a single location, the uncertainty regarding COVID-19 in children extends to its pathogenesis, presentation, clinical course, treatment, and prognosis. Important questions with medical, sociological, and economic implications therefore remain unanswered or incompletely resolved. Of particular interest is also the emergence of a systemic hyperinflammatory syndrome that is Kawasaki-like in presentation [5,6,7,8,9]. Starting in the late spring of 2020, a Kawasaki-like disease linked to COVID-19 was increasingly noted in parts of Europe and the United States. Known as multisystem inflammatory syndrome in children (MIS-C), this post-infectious disease with an onset between 2–4 weeks after the infection occurred in children aged between 6 months and 17 years [10]. This syndrome is of particular concern, as most cases have been reported in children who were previously healthy with no underlying medical conditions [11].

**Figure 1:**
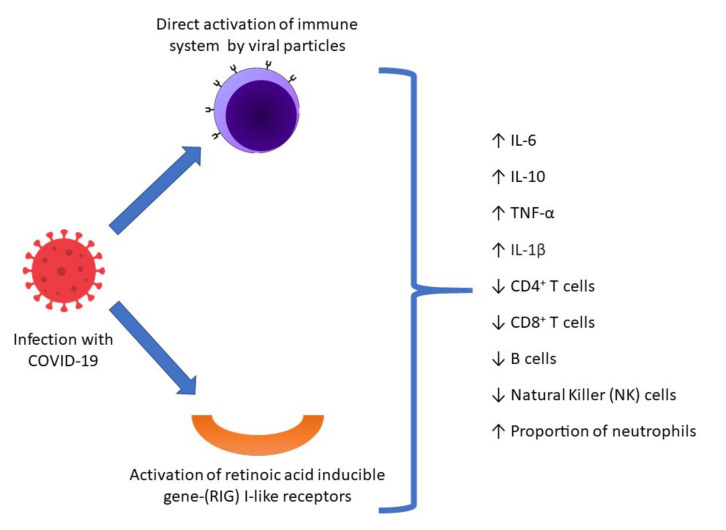
Hyperimmune response in COVID-19. COVID-19 may trigger a dual immune response, both through direct activation of the immune system by viral particles and by activation of the immune-promoting retinoic acid inducible gene-(RIG) I-like receptors (RLRs) and subsequent release of retinoic acid and retinyl esters into circulation. The hyperimmune response in COVID-19, characterized by elevated inflammatory markers and lymphopenia, particularly of cytotoxic lymphocytes, and relative preponderance of neutrophils, may contribute to the pathophysiology of MIS-C.

Although exact mechanisms are not fully understood, the pathophysiology of MIS-C hinges upon multilayered hyperinflammation due to COVID-19 infection. As suspected, COVID-19 viral particles trigger an immune response. Furthermore, an additional immune response may be due to activation of the immune-promoting retinoic acid inducible gene-(RIG) I-like receptors (RLRs) [12]. It has been suggested that release of hepatic retinoic acid and retinyl esters into circulation may lead to end-organ damage throughout the body [13]. Such widespread distribution of toxic substrates would produce a spectrum of clinical outcomes. The cumulative effect of these cascades may promote an “out-of-control” hyperimmune response [14] (Figure 1). Indeed, studies have demonstrated the presence of elevated levels of inflammatory factors such as interleukin-6 (IL-6), tumor necrosis factor-α (TNF-α), and IL-1β, in patients with COVID-19 [15]. In more severely ill patients, lymphopenia may be present due to exhaustion, specifically, of cytotoxic lymphocytes [16,17]. The reported systemic hyperinflammation in patients, likely due to the presence of pro-inflammatory cytokines, has been associated with vascular leakage, multiorgan damage, and death [18]. Such dysregulation of the immune response may underlie the emergence of the MIS-C presentation.

From previous studies [19], it is increasingly evident that the manifestations of COVID-19 in children are different than those of adults, while at the same time reflecting heterogeneous presentations among infected children. This heterogeneity compounds the difficulty with accurately and definitively presenting a clear evidence-based understanding of COVID-19. The lack of expansive pediatric COVID-19 data from unified populations coupled with the diversity of clinical presentation supports the utility of data reflecting the collective experience. This systematic review aims to collate a collective clinical information on disease characteristics and course, systemic symptoms and complications, and diagnostic testing of COVID-19 in children to assess its full clinical spectrum.

## 2. Materials and Methods

**Figure 2:**
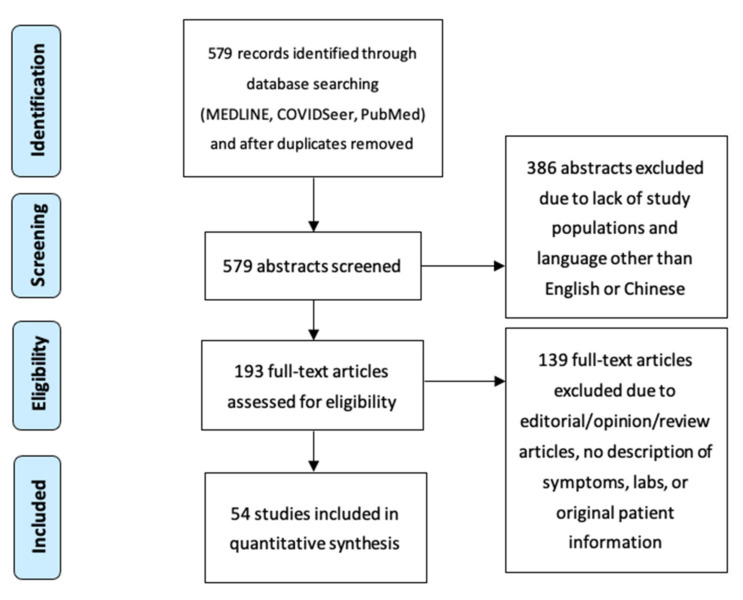
Preferred Reporting Items for Systematic Reviews and Meta-Analyses (PRISMA) flow chart illustrating the inclusion and exclusion of studies for the review [20]. Published under the terms of the Creative Commons Attribution License.

An electronic literature search was performed using the databases COVIDSeer (Pennsylvania State University, University Park, PA, USA), MEDLINE (Mundelein, IL, USA), and PubMed (National Center for Biotechnology Information, National Library of Medicine, Bethesda, MD, USA) for English and Chinese language articles from 1 January 2020 through 1 March 2021. Chinese articles were read by a physician fluent in both English and Chinese. Articles were selected for using Preferred Reporting Items for Systematic Reviews and Meta-Analyses (PRISMA) guidelines (Figure 2). Each article was screened independently by a reviewer. Keywords for the search were pediatric cases, COVID-19, child, coronavirus infections/epidemiology, pandemics, pneumonia, viral/epidemiology, United States/epidemiology, and Kawasaki disease. Screened and included articles were case reports, case series, systematic reviews, and prospective studies from single or multiple health centers. Patients were ages 30 days–18 years old with symptomatic and/or laboratory confirmed cases of COVID-19 in a hospital setting, depending on the study. All included articles used real-time polymerase chain reaction (PCR) of nasopharyngeal secretions as the test of choice.

Our initial search produced 579 records, all of which were screened for inclusion. Screening excluded any records unavailable in English or Chinese, as well as any records that did not identify the study population. The remaining 193 records were then further examined for eligibility. Inclusion criteria for articles necessitated the presence of full article text, presence of the laboratory/imaging data (if part of the study), and the listing/categorization of relevant symptoms. In particular, documentation of the presence or absence of computed tomography (CT) chest abnormalities, intensive care unit (ICU) admission, low oxygen saturations (SpO_2_%), cerebrospinal fluid (CSF) abnormalities, lymphopenia, and elevated liver function tests (LFTs) were required.

For cases in which there was no recorded laboratory confirmation of COVID-19, the article was only included if patients had primary symptoms of COVID-19 that fulfilled Centers for Disease Control and Prevention guidelines for diagnosis: fever, cough, with or without myalgias and sore throat, as well as bilateral ground glass opacities or unilateral consolidation on CT of the chest [6]. While 139 records were excluded, 54 articles were included for full evaluation as part of the review (Table 1). Duplicated articles published using the same population were treated as one set of data only. While many of the articles were case reports or series of pediatric cases for COVID-19, review articles on COVID-19 in children were also included. These cohesive reviews that do not include the articles used in this study served to strengthen the evaluation of the clinical data that was extracted. Each article was reviewed by one researcher, and data was collected independently by reviewers and organized in a shared Excel file. The studies considered patients in China (24/54), other Asian countries (3/54), Turkey (4/54), Europe (13/54), North America (9/54), and South America (1/54).

The 54 articles contained information from a total of 4811 children, 4268 (88.71%) of whom presented with typical COVID-19 and 543 (11.29%) of whom presented with MIS-C. For each article, we noted how many individuals in each diagnostic category exhibited the following general characteristics: male gender, fever, sore throat, rash, fatigue, myalgia/malaise, shortness of breath, cough, nausea/vomiting, diarrhea, ICU admission, septic shock, lymphocytopenia, abnormal CT scan of the chest, pneumonia, and elevated liver function. We also recorded the presence of the following neurological symptoms: headaches, syncope, focal deficits, dizziness, anosmia/hypogeusia, altered mental state, seizures, sleep disturbance, and stroke.

The ideal analysis would have started with a model that included all or multiple characteristics/symptoms, so as to observe potential relationships among them. However, the data in our source articles were often presented in aggregate, meaning that we did not have a full profile of all variables for every individual in the study. As such, we analyzed the association of each variable with typical COVID-19 and MIS-C presentation using a series of binary logistic regressions. In terms of general characteristics, we removed pneumonia and elevated liver function from the analyses due to the rarity of these conditions in the individuals with MIS-C. All neurological symptoms other than headache were excluded for the same reason. We calculated z-scores, p values, odds ratios, and 95% confidence intervals for the odds ratios for the remaining characteristics and symptoms using Stata/SE v16 (Stata Corp LLC, College Station, Texas, USA).

## 3. Results

### 3.1. All Cases

The first aim of this study was to identify clinical trends and recurring phenotypic manifestations of the novel coronavirus in children. In this context, the individual case studies offered cohesive trends but also clearly highlighted the heterogeneity of outlier cases. Of this group, 2277 out of 4811 cases, where data was available, were males (47.33%). Additionally, 2928 out of 4811 cases were inpatient (60.86%). Importantly, this sample set included a wide variety of asymptomatic, mildly symptomatic, and critical cases, positioning the symptomatic nature of pediatric COVID-19 as two extremes. The review focused primarily on the clinical features of these cases. For the total number of pediatric manifestations of COVID-19 (*n* = 4811), the most frequently occurring symptoms were fever (50.84%) and sore throat (31.78%). Respiratory symptoms, namely shortness of breath (26.07%) and pneumonia (22.55%), were the next most frequently reported symptoms. Children with COVID-19 displayed nausea and/or vomiting 9.1% of the time and diarrhea 8.65% of the time. Generic viral symptoms such as rash (2.31%) and fatigue (2.93%) were all relatively rare. Neurologic symptoms, including headache (8.71%), seizure (0.21%), dizziness (0.10%), altered mental status (0.19%), sleep disturbance (0.06%), focal neurologic deficits (0.19%), and syncope (0.02%), were relatively rare. Similarly, anosmia—although widely reported in the adult population—was reported in only 3.24% of all pediatric cases.

Laboratory findings were remarkable for lymphopenia in 6.15% of cases. Liver function tests were elevated in 31/4811 cases (0.64%). While only 622 of the 4811 (12.93%) patients had CT of the chest, 339 of those (54.50%) were abnormal. Out of all the reported patients, 429 (8.92%) were admitted to the ICU. Septic shock occurred in 130 patients (2.70%).

### 3.2. Kawasaki-like Multisystem Inflammatory (MIS-C) Cases

Out of the total reported 4811 patients in this review, 543 (11.28%) had presentations consistent with MIS-C. Of these, 59.67% were male. Fever was nearly ubiquitous, with 530 (97.61%) reporting fever. Gastrointestinal symptoms, such as nausea/vomiting (57.27%) and diarrhea (53.22%), were common. Rash was not uncommon (19.15%) among children with MIS-C, while respiratory symptoms such as cough (13.63%), shortness of breath (11.23%), chest tightness (2.03%), and pneumonia (0.18%) were less common. Neurologic symptoms, namely altered mental status (1.29%), seizures (0.37%), syncope (0.18%), and stroke (0.18%), were rare. Headaches were the most common neurologic symptom, being reported in 13.81% of cases.

On laboratory workup, less than half of the patients (42.54%) had lymphopenia. Only one patient had elevated LFTs. Of the 543 patients with MIS-C, 100% were inpatient, 67.22% were admitted to the ICU, and 21.36% had septic shock.

### 3.3. Children without MIS-C

Of those 4268 patients without MIS-C, 1953 (45.76%) were male. The most common symptoms included fever (44.89%), along with sore throat (35.43%), shortness of breath (27.95%) and pneumonia (25.40%). However, cough (18.67%) was less common. Gastrointestinal symptoms, such as nausea/vomiting (2.98%) and diarrhea (2.98%), were even less common. Rash was scarce (0.16%). Neurologic symptoms, namely anosmia (3.66%), seizures (0.19%), dizziness (0.12%), altered mental status (0.05%), sleep disturbance (0.05%), and stroke (0.05%) were likewise rare. Headache was the most common neurologic symptom (8.06%). Syncope was not reported in any cases.

On laboratory workup, few of the patients (1.52%) had lymphopenia, and 30 patients (0.70%) had elevated LFTs. Of the 4268 patients without MIS-C, 68.60% were inpatient, 1.50% were admitted to the ICU, and 0.33% had septic shock.

### 3.4. Comparing Children with and without MIS-C

In terms of general characteristics, sore throat, shortness of breath, and cough were more associated with a typical presentation of COVID-19 than MIS-C (Table 2). All other variables (male sex, fever, rash, fatigue, myalgia/malaise, nausea/vomiting, diarrhea, ICU admission, septic shock, lymphocytopenia, and abnormal CT scan of the chest) were more associated with MIS-C than with typical COVID-19 presentation (Table 2).

In terms of neurological symptoms, headache showed a greater association with MIS-C than typical COVID-19 presentation (Table 2).

## 4. Discussion

This study was able to achieve several objectives. First, by examining a breadth of reported cases across both the English language and Chinese language literature, a more diverse clinical perspective was achieved. In doing so, a more comprehensive evaluation of COVID-19 in children was developed. With a slight preponderance of females, there was no clear overall gender predilection for COVID-19 infections (overall) in children. Fever was the most common symptom across all pediatric populations, although it was not ubiquitous. Interestingly, two of the symptoms commonly heralded as “classic” for COVID-19 (cough and anosmia) were rare in children. Anosmia in particular was reported in only 156 of the cases despite being one of the most common symptoms of COVID-19 in adults [72]. While this could be due to reporting bias, the virtually non-existent reporting of anosmia across numerous studies suggests that this lack of anosmia in children with COVID-19 is a noticeable pattern. Such differences could be due to intrinsic differences in the olfactory neuroepithelium between children and adults, but further research is required to elucidate this difference [73]. Similarly, other generic viral symptoms (such as rash and fatigue) were also rare in this cohort of children. Respiratory symptoms (such as sore throat, shortness of breath, and pneumonia) were the most common symptoms besides fever, but they were all present in less than 50% of the pediatric population. In summary, COVID-19 in children presents commonly (though not always) with fever. Anosmia does not appear to be prevalent in pediatric COVID-19.

Furthermore, neurological symptoms were overall rare in children. A significant presentation of COVID-19 in adults is neurological symptoms, including headache, stroke, and impaired consciousness, which are related to elevated pro-inflammatory cytokines [74,75,76,77,78].

By examining symptoms across the clinical spectrum of COVID-19, this study was able to examine patterns that distinguish those children with a milder clinical course from those with the fulminant inflammatory syndrome known as MIS-C. The odds ratios resulting from this study were robust, implying that symptoms with a high predictive value were identified. Male gender is modestly associated with MIS-C. Inflammatory symptoms such as myalgia/weakness, fever, and rash were strongly associated with MIS-C. Such an observation may be explained by the fact that MIS-C bears similarity to cytokine storms in adults who have COVID-19 infection [79]. Gastrointestinal symptoms were common in MIS-C, while respiratory symptoms seem to be more representative of the typical course of COVID-19. These findings could allow for stratification of children with COVID-19. Aside from noting which symptoms show increased association with MIS-C, the absence of such symptoms could allow for reassurance that the possibility of MIS-C is low.

There are several limitations to our study that arise from the nature of the study. In a review, the data are the resultant of previous reports and so the likelihood of a report-bias is necessarily increased. In our study, this stands out in the high proportion of children with MIS-C. While 11.29% of the patients in this study had MIS-C, the overall incidence of MIS-C is estimated at between 0.016% and 0.31% of infected children [10]. This over-representation of MIS-C cases arises due to the reported cases being largely from an inpatient medical facility setting, to where the MIS-C cases would more likely be bottle-necked. Furthermore, children with exceedingly mild cases or even asymptomatic presentations would be unlikely to present to a physician and therefore not be reported in studies, thus these results may not represent general population-based COVID-19 presentation in children.

Additionally, the ability to cull data for specific symptoms (for example, the number of patients with anosmia) was reliant on whether an individual study specifically queried for that symptom. While, as is the pattern of most review articles, a lack of mentioning a symptom is considered a negative finding (that is, that the symptom is not present), the reality could be that the question was simply not asked. As such, the presence of symptoms (particularly mild symptoms, such as fatigue) could be underrepresented.

Furthermore, the issue of variants of the COVID-19 virus has become an increasing concern as the pandemic stretches on. Each variant carries with it a particular transmissibility, lethality, and clinical nuance. In a retrospective study accumulating data before variant subtypes were considered, the fractionation of the data by variant is not possible. In areas and periods of time with a particular variant predominance, the clinical presentation of the virus may be quite different than what is presented in this study.

Nonetheless, this systematic review provides valuable insight into the presentation of COVID-19 in children. Understanding the manifestations of the virus in a pediatric population is critical, as although outcomes are generally less severe compared to the adult population, children and adolescents can transmit the virus to populations, such as the elderly, who are more susceptible to severe complications [3]. Furthermore, as more children return to school and pandemic restrictions are relaxed, understanding the presentation of COVID-19 and which populations are more suspectable to intensive care unit admission and developing MIS-C is important for planning a safe return to in-person learning, especially because many in this population are not yet eligible for vaccination against the virus. Additionally, it is critical to focus attention on groups who are more susceptible to contracting COVID-19, such as children with developmental disabilities and/or respiratory support, who may have difficulty tolerating face masks or whose behavior (such as mouthing objects) predisposes them to contracting the virus [80,81]. Future research should focus on which populations are most vulnerable to infection by SARS-CoV-2 and developing MIS-C, what policies are in place for the return to school of vulnerable children with pre-existing and chronic conditions, and what support resources are available for these children. Lastly, the effects of different variants of SARS-CoV-2 on children should also be investigated. Although some variants, such as the Variant of Concern (VOC) 202012/01 (lineage B.1.1.7) originating from the United Kingdom, have been found to be more infectious and deadly in adults, limited research exists in the clinical presentation and outcomes of different SARS-CoV-2 variants in children [82].

## 5. Conclusions

This systematic review provides meaningful insight into the spectrum of clinical manifestation of COVID-19 in children, especially with and without MIS-C. Fever, sore throat, shortness of breath, and pneumonia were the most common symptoms across both groups, whereas cough and anosmia were rare. Symptoms associated with inflammation, such as myalgia/weakness, fever, and rash markedly increase the risk of MIS-C. These results shed light on investigations of the pathophysiology of the virus in the pediatric population. Furthermore, the data demonstrating risk factors of development of MIS-C could allow for risk stratification of children with COVID-19 based upon clinical presentation. Future studies can build upon this information by investigating the lack of cough and anosmia in children, which is potentially due to differences in the olfactory neuroepithelium between children and adults. Additionally, future investigations should focus on pediatric populations vulnerable to COVID-19, and what resources and policies are in place as children return to in-person learning.

## Figures and Tables

**Table 1:** Characteristics of the studies included in the review.

Study	Publication Date	Study Location	Sample	Population
Dong et al. [1]	April 2020	China	728	Inpatients with suspected and laboratory confirmed coronavirus disease 2019 (COVID-19)
Cui et al. [21]	March 2020	China	1	Pediatric patients admitted to hospital with confirmed COVID-19
Li et al. [22]	March 2020	China	2	Pediatric patients admitted to hospital with confirmed COVID-19
Su et al. [23]	February 2020	China	9	Children admitted to infectious disease hospital for COVID-19
Ji et al. [24]	March 2020	China	2	Pediatric patients admitted to hospital with confirmed COVID-19
Sun et al. [25]	September 2020	China	8	Intensive care unit (ICU) patients with confirmed COVID-19
Dugue et al. [26]	April 2020	North America (United States)	1	A 6-week-old male infant with confirmed COVID-19, inpatient
Lu et al. [27]	March 2020	China	171	Children with COVID-19 at Wuhan Children’s Hospital, inpatient
Cai et al. [28]	February 2020	China	10	Inpatients with confirmed COVID-19
Kam et al. [29]	February 2020	Asia (Singapore)	1	Confirmed pediatric case, inpatient
Chen et al. [30]	April 2020	China	12	Inpatients with laboratory confirmed COVID-19
Zhang et al. [31]	March 2020	China	2	Twin girls, inpatient
Zhang et al. [32]	March 2020	China	1	3-month-old girl, inpatient
Wang et al. [33]	April 2020	China	31	24 inpatient and 7 outpatient with confirmed COVID-19
Feng et al. [34]	April 2020	China	15	Pediatric patients admitted to hospital with confirmed COVID-19
Chen et al. [35]	February 2020	China	1	Pediatric patient admitted to hospital with confirmed COVID-19
Jiehao et al. [36]	February 2020	China	1	Pediatric patient admitted to hospital with confirmed COVID-19
Xing et al. [37]	March 2020	China	3	Pediatric patient admitted to hospital with confirmed COVID-19
Wei et al. [38]	February 2020	China	9	Pediatric patients admitted to hospital with confirmed COVID-19
Riphagen et al. [7]	May 2020	Europe (England)	8	8 children with hyperinflammatory shock, inpatient
Mao et al. [39]	May 2020	China	1	Pediatric patient admitted to hospital with confirmed COVID-19
See et al. [40]	March 2020	Asia (Malaysia)	4	Pediatric patients admitted to hospital with confirmed COVID-19
Paret et al. [41]	July 2020	North America (United States)	2	Pediatric patients admitted to hospital with confirmed COVID-19
Qui et al. [42]	April 2020	China	1	Hospitilalized infant with failure to thrive and confirmed COVID-19
Gefen et al. [43]	May 2020	North America (United States)	1	Child with history of autism and obstructive sleep apnea with labratory confirmed COVID-19, inpatient
Giacomet et al. [44]	April 2020	Europe (Italy)	1	Infant admitted for severe laboratory confirmed COVID-19
Musolino et al. [45]	April 2020	Europe (Italy)	10	Pediatric patients admitted to hospital with confirmed COVID-19
Qiu et al. [46]	March 2020	China	36	Pediatric patients admitted to hospital with confirmed COVID-19
Denina et al. [47]	June 2020	Europe (Italy)	8	Pediatric patients admitted to hospital with confirmed COVID-19
Oualha et al. [48]	June 2020	Europe (France)	27	Patients admitted to pediatric ICU/high-dependency unit with severe COVID-19
Jiang et al. [49]	April 2020	China	2	Children with COVID-19 coinfected with human respiratory viruses and Mycoplasma pneumoniae
Pinar Senkalfa et al. [50]	June 2020	Turkey	45	Patients with cystic fibrosis and COVID-19, inpatient
Bapst et al. [51]	June 2020	Europe (Switzerland)	1	Previously healthy 13-year-old boy with fever of 7 days, inpatient
Bai et al. [52]	July 2020	China	25	Pediatric patients admitted to hospital with confirmed COVID-19
De Ioris et al. [53]	May 2020	Europe (Italy)	22	Pediatric patients admitted to hospital with confirmed COVID-19
Haslak et al. [54]	July 2020	Turkey	404	Confirmed COVID-19 cases, patients with contact history, or symptoms suggestive of COVID-19 (all with autoinflammatory diseases). 24 inpatient and 380 outpatient
Türe et al. [55]	November 2020	Turkey	24	Emergeny department admits with confirmed COVID-19
Tran et al. [56]	September 2020	Europe (France)	1	Pediatric patients admitted to hospital with confirmed COVID-19
Zhang et al. [57]	June 2020	China	34	Pediatric patients admitted to hospital with confirmed COVID-19
Korkmaz et al. [58]	June 2020	Turkey	81	44 inpatient and 37 outpatient with confirmed COVID-19
King et al. [59]	January 2021	North America (Canada)	1987	8 inpatient and 1979 outpatient with confirmed COVID-19
Garcia-Howard et al. [60]	August 2020	Europe (Spain)	1	Pediatric patient admitted to hospital with confirmed COVID-19
Zhang et al. [61]	July 2020	China	534	Outpatient with confirmed COVID-19
Dufort et al. [62]	July 2020	North America (United States)	191	Patients admitted to hospital with COVID-19 and suspected multisystem inflammatory syndrome (MIS-C)
Deza Leon et al. [63]	May 2020	North America (United States)	1	Previously healthy child with sore throat, fever, reduced oral intake 6 days before admission
Chiotos et al. [64]	May 2020	North America (United States)	6	Critically ill children with multisystem inflammatory syndrome, inpatient
Feldstein et al. [10]	July 2020	North America (United States)	186	Patients with serious illness leading to hospitalization, an age of less than 21 years, fever that lasted for at least 24 h, laboratory evidence of inflammation, multisystem organ involvement, and evidence of COVID-19
Jones et al. [65]	April 2020	North America (United States)	1	Infant admitted due to severe presentation of COVID-19
Oberweis et al. [66]	July 2020	Europe (Luxembourg)	1	Hospital admission of a previously healthy child with 4-day fever, coughing, weight loss, fatigue
Toubiana et al. [67]	December 2020	Europe (France)	23	Cases with fever admitted to hospital
García-Salido et al. [68]	November 2020	Europe (Spain)	74	Intensive care unit patients with a diagnosis of COVID-19 and patients who met the case definition for MIS-C
Lima-Setta et al. [69]	November 2020	South America (Brazil)	56	Patients admitted to the ICU with COVID-19
Vukomanovic et al. [70]	October 2020	Europe (Serbia)	3	Pediatric patients admitted to hospital with confirmed COVID-19
Navaeifar et al. [71]	January 2021	Asia (Iran)	1	Patients admitted to the ICU with COVID-19

**Table 2:** Results from the logistic regressions, with odds ratios (OR) and confidence intervals (CI), for children with MIS-C and without MIS-C (typical) [1,7,10,21,22,23,24,25,26,27,28,29,30,31,32,33,34,35,36,37,38,39,40,41,42,43,44,45,46,47,48,49,50,51,52,53,54,55,56,57,58,59,60,61,62,63,64,65,66,67,68,69,70,71].

	Yes				No								
	MIS-C		Typical		MIS-C		Typical					OR 95% CI	
General Characteristics	*n*	%	*n*	%	*n*	%	*n*	%	z	*p*	OR	Lower	Upper
Male	324	14.2	1953	85.8	219	8.6	2315	91.4	6.06	<0.001	1.75	1.46	2.1
Fever	530	21.7	1916	78.3	13	0.5	2352	99.5	13.86	<0.001	50.05	28.77	87.05
Sore throat	17	1.1	1512	98.9	526	16.0	2756	84.0	−11.4	<0.001	0.06	0.04	0.1
Rash	104	93.7	7	6.3	439	9.3	4261	90.7	12.63	<0.001	144.21	66.66	311.95
Fatigue	39	27.7	102	72.3	504	10.8	4166	89.2	5.93	<0.001	3.16	2.16	4.62
Myalgia/malaise	53	36.8	91	63.2	490	10.5	4177	89.5	8.94	<0.001	4.96	3.49	7.05
Shortness of breath	61	4.9	1193	95.1	482	13.6	3075	86.4	−8	<0.001	0.33	0.25	0.43
Cough	74	8.5	797	91.5	469	11.9	3471	88.1	−2.86	0.004	0.69	0.53	0.89
Nausea/vomiting	311	71.0	127	29.0	232	5.3	4141	94.7	30.2	<0.001	43.71	34.21	55.85
Diarrhea	289	69.5	127	30.5	254	5.8	4141	94.2	29.01	<0.001	37.1	29.06	47.36
ICU admission	365	85.1	64	14.9	178	4.1	4204	95.9	31.5	<0.001	134.7	99.28	182.74
Septic shock	116	89.2	14	10.8	427	9.1	4254	90.9	15.35	<0.001	82.55	46.99	145
Lymphocytopenia	231	78.0	65	22.0	312	6.9	4203	93.1	25.42	<0.001	47.87	35.53	64.51
Abnormal CT-Chest	97	28.6	242	71.4	446	10.0	4026	90.0	9.88	<0.001	3.62	2.8	4.67
Pneumonia	1	0.1	1084	99.9	542	14.5	3184	85.5					
Elevated liver function test	1	3.2	30	96.8	542	11.3	4238	88.7					
Neurological symptoms													
Headaches	75	17.9	344	82.1	468	10.7	3924	89.3	4.42	<0.001	1.83	1.4	2.39
Syncope	1	100.0	0	0.0	542	11.3	4268	88.7					
Focal deficits	0	0.0	9	100.0	543	11.3	4259	88.7					
Dizziness	0	0.0	5	100.0	543	11.3	4263	88.7					
Anosmia/hypogeusia	0	0.0	156	100.0	543	11.7	4112	88.3					
Altered mental status	7	77.8	2	22.2	536	11.2	4266	88.8					
Seizures	2	20.0	8	80.0	541	11.3	4260	88.8					
Sleep disturbance	1	33.3	2	66.7	542	11.3	4266	88.7					
Stroke	1	33.3	2	66.7	542	11.3	4266	88.7					

Yes and no refer to the presentation of characteristics/symptoms. MIS-C-multisystem inflammatory syndrome in children; ICU-intensive care unit; CT-computed tomography.

## Data Availability

Data can be requested from the corresponding author. The review protocol can likewise be requested from the corresponding author.
